# Timing of outbreak-triggered unit-wide screening and COVID-19 outbreak size in long-term care and related social welfare facilities in Okinawa, Japan

**DOI:** 10.1016/j.ijregi.2026.101042

**Published:** 2026-10-13

**Authors:** Yining S. Xu, Kuniko Takahashi, Yusuke Shimakawa, Takashi Nakamura, Toru Itokazu, Yoshihiro Takayama, Kenji Mizumoto

**Affiliations:** 1 Graduate School of Advanced Integrated Studies in Human Survivability, Kyoto University, Kyoto, Kyoto, Japan; 2 Emergency Medical Center, Urasoe General Hospital, Urasoe, Okinawa, Japan; 3 The Okinawa Prefecture Commission for Epidemiological and Statistical Analysis, Naha, Okinawa, Japan; 4 Unité d’Epidémiologie des Maladies Emergentes, Institut Pasteur, Université Paris Cité, Paris, France; 5 Pasteur International Unit at Kumamoto University/Japan Institute for Health Security, Tokyo, Japan; 6 Joint Research Center for Human Retrovirus Infection, Kumamoto University, Kumamoto, Japan; 7 Department of Emergency Medicine, Nakagami Hospital, Okinawa, Okinawa, Japan; 8 Okinawa Prefectural Government, Naha, Okinawa, Japan; 9 Division of Infectious Diseases, Okinawa Prefectural Chubu Hospital, Uruma, Okinawa, Japan

**Keywords:** COVID-19, Long-term care facilities, Social welfare facilities, Outbreak, Infection control, Japan

## Abstract

**Objectives:**

Coronavirus disease 2019 outbreaks in long-term care facilities can spread rapidly, but evidence on whether delayed outbreak-triggered unit-wide screening is associated with outbreak size is limited.

**Methods:**

We conducted a retrospective observational study using outbreak-support records from long-term care and related social welfare facilities in Okinawa Prefecture, Japan, from April to September 2022. We included outbreaks with documented unit-wide screening. The outcome was the final outbreak size. The exposure was the delay from outbreak recognition to the first unit-wide screening (same day, 1 day, 2 days, or ≥3 days). We used multivariable negative binomial regression, adjusting for index case type, facility type, recognition period, and facility-level resident vaccination coverage.

**Results:**

We analyzed 707 outbreaks. Compared with same-day screening, the adjusted incidence rate ratios for outbreak size were 0.95 (95% confidence interval 0.72-1.26) for a 1-day delay, 1.16 (0.87-1.54) for a 2-day delay, and 1.44 (1.09-1.91) for delays of ≥3 days. The association was attenuated after adjustment for on-site infection prevention and control visit timing.

**Conclusion:**

Delays of ≥3 days from outbreak recognition to the first unit-wide screening were associated with a larger final outbreak size. Earlier outbreak-triggered screening may support outbreak control in long-term care facilities, although causal interpretation is limited by the observational design.

## Highlights

• We analyzed 707 coronavirus disease 2019 outbreaks in long-term care facilities and related social welfare facilities.
• Unit-wide screening ≥3 days after recognition was associated with larger outbreaks.
• The ≥3-day estimate attenuated after adjustment for on-site infection prevention and control timing.
• Estimates were similar across approaches accounting for within-facility correlation.
• Prompt outbreak-triggered screening may support outbreak control in long-term care facilities.

## Introduction

Long-term care facilities (LTCFs) have borne a disproportionate coronavirus disease 2019 (COVID-19) burden because residents are older, have multiple comorbidities, and depend on close-contact care [[1], [2], [3], [4]]. In Japan, clusters in long-term care hospitals and facilities contributed substantially to severe cases and deaths, whereas repeated severe acute respiratory syndrome coronavirus 2 waves in Okinawa strained limited bed capacity and emergency transport [3,5]. Preventing large outbreaks in LTCFs and other social welfare facilities is, therefore, essential [[1], [2], [3], [4],^6^].

Presymptomatic and asymptomatic transmission is common in LTCFs [[7], [8], [9], [10]]. Facility-wide and serial testing can identify otherwise undetected infections and support outbreak control, particularly with frequent testing and rapid turnaround [5,[7], [8], [9], [10], [11], [12], [13], [14], [15], [16]]. International guidance, therefore, recommends prompt outbreak-response testing, with broader unit- or facility-wide testing when indicated [12,13,17].

Previous studies have linked delayed outbreak identification or completion of facility-wide testing with a greater outbreak burden [12,18]. These studies assessed different aspects of timeliness, including the interval from first-case identification to completion of facility-wide testing [12] and cumulative infectious resident-days at outbreak identification [18]. Neither study examined the interval from operational outbreak recognition to the first outbreak-triggered unit-wide screening during the Omicron period.

Okinawa Prefecture operated an outbreak-support program that provided infection prevention and control (IPC) advice and outbreak testing, alongside voluntary staff reverse transcription polymerase chain reaction (RT-PCR) screening every 2-3 weeks [6]. In a related questionnaire study, outbreaks were smaller when staff index cases were detected through contact-based rather than routine screening; however, the study did not examine the timing of unit-wide screening after outbreak recognition [6].

Because the timing of unit-wide screening after outbreak recognition may vary according to outbreak circumstances and operational capacity, we conducted an outbreak-level observational study using routinely collected support records from social welfare facilities in Okinawa Prefecture. Our aim was to examine whether the interval from outbreak recognition to the first unit-wide screening was associated with final outbreak size among supported facility outbreaks.

## Methods

### Study design and data source

We conducted a retrospective observational study using a line list of supported COVID-19 outbreaks in long-term care and related social welfare facilities in Okinawa Prefecture, Japan. The line list was maintained by the prefectural outbreak-support team and included facility characteristics, outbreak timelines, and final outbreak size. These records were generated through a prefectural outbreak-support program that provided remote and, when needed, on-site IPC advice and coordinated additional testing during outbreaks. For this analysis, we included outbreaks recognized between April 1 and September 30, 2022, when Omicron variants predominated. Because the data set was assembled for operational response rather than research, some variables had missing or limited details.

### Setting and outbreak-support system

Okinawa Prefecture operated a support program for social welfare facilities that provided remote and on-site IPC guidance and coordinated additional testing during outbreaks [6]. When facility managers recognized a suspected outbreak—typically one or more confirmed cases with concern for within-facility spread—they notified the outbreak-support team or the local public health center. The team reviewed zoning and cohorting plans and coordinated outbreak-related testing. Prefectural guidance recommended initiating polymerase chain reaction testing within 24 hours after a positive case was recognized, including potentially exposed residents and staff beyond formally designated close contacts; broader floor- or facility-wide testing and repeat testing after 5-7 days were recommended when indicated [19]. The 24-hour recommendation applied to initial testing, not completion of unit-wide screening; no uniform mandatory interval was specified for the first unit-wide screening, the timing and scope of which varied according to outbreak circumstances and operational capacity.

In parallel, a voluntary RT-PCR screening program offered staff self-collected saliva testing every 2-3 weeks [6]. This program was distinct from outbreak-triggered unit-wide screening, although a positive result could prompt outbreak recognition and subsequent response testing. Information sufficient to determine the index case detection pathway and the timing of recent routine staff screening was not consistently available for individual outbreak episodes in the analytic data set.

### Eligibility criteria

The source data set contained COVID-19 outbreak episodes in social welfare facilities that were recorded by the prefectural outbreak-support system between April 1 and September 30, 2022. For the present analysis, we included outbreak episodes with a documented date of first unit-wide screening on or after outbreak recognition and a defined final outbreak size. Accordingly, inference is most directly applicable to outbreaks captured within this prefectural support system. Because the present analysis required a documented unit-wide screening, the analytic sample represents a subset of facility outbreaks captured by the public health response system, and we could not formally assess its representativeness relative to all facility outbreaks in Okinawa during the study period.

### Outcome and main exposure

The primary outcome was the final outbreak size, defined as the total number of laboratory-confirmed COVID-19 cases among residents and staff in each outbreak, including the index case. The main exposure was the delay from outbreak recognition to the first unit-wide screening, categorized as same-day, 1 day later, 2 days later, or ≥3 days later. Outbreak recognition was defined operationally as the date on which the outbreak was recognized and referred to the prefectural support system rather than the date of the first transmission within the facility. Therefore, the exposure reflects the timeliness of screening after the operational recognition of an outbreak and should not be interpreted as the time from the true outbreak onset.

### Covariates

Covariates were selected *a priori* as potential confounders of the association between screening delay and outbreak size. Index case type was classified as staff vs resident or non-staff (including residents, day-service users, family members, and episodes with unknown route of introduction), with staff as the reference group. Facility type, recorded according to Japanese regulatory categories, was grouped into six categories: special nursing homes for the elderly, long-term care health facilities, fee-based homes for older adults, service housing for older people, other small elderly facilities (group homes for older adults, small-scale multifunctional in-home care facilities, and care houses), and other or unknown facilities, which also included disability welfare facilities. In the primary analysis, disability welfare facilities were included in the other/unknown category and examined separately in sensitivity analyses.

Facility-level resident vaccination coverage was defined as the percentage of residents who had received at least two doses of the COVID-19 vaccine at the time of the outbreak and was categorized as ≥80%, 50%-79%, <50%, and unknown or not ascertained (reference: ≥80%). The unknown or not ascertained category was retained to preserve sample size but its coefficient was interpreted cautiously because it may reflect differences in documentation practices or facility reporting processes rather than vaccination status.

To capture temporal changes in community incidence and circulating variants, we grouped the month of outbreak recognition into April-May, June-July and August-September 2022 (reference: April-May). The outbreak-support team could provide on-site IPC visits in addition to remote support; for each outbreak, we classified the timing of the first on-site visit as no on-site visit, visit within 0-1 days after recognition, visit ≥2 days after recognition, or unknown/missing (reference: no on-site visit). Because on-site visits were more likely to be provided for larger or more complex outbreaks, this variable was treated as a sensitivity analysis covariate rather than a causal exposure.

### Statistical analysis

We described outbreak characteristics overall and by screening-delay category and index case type, reporting medians with interquartile ranges (IQRs) and means for outbreak size. For outbreaks classified as having a delay of ≥3 days, we additionally calculated the interval in calendar days as the difference between the recorded screening and recognition dates and summarized its median, IQR, range, and frequency distribution. We also cross-tabulated resident vaccination coverage categories by facility type, reporting counts and row percentages. Vaccination coverage missingness by facility type was compared using Pearson’s chi-square test.

We then modeled the final outbreak size using negative binomial regression because the outcome was an overdispersed count. The primary multivariable model adjusted for index case type, facility type, month of outbreak recognition (April-May, June-July, and August-September), and facility-level resident vaccination coverage. For the primary model, 95% confidence intervals (CIs) and two-sided *P*-values were calculated using heteroskedasticity-robust HC1 standard errors. Because facility-level denominators, such as bed capacity or the number of residents and staff at risk, were unavailable in the outbreak-support records, the outcome was analyzed as an absolute case count rather than an attack rate or standardized incidence measure. We, therefore, considered residual confounding by facility size to be likely and interpreted the estimates as associations with larger absolute outbreak size.

Sensitivity analyses used a six-category month variable (April-September), treated disability welfare facilities as a separate facility type category, included a model with a log-offset of 1.5 × category-specific average licensed capacity as a proxy for the population at risk, and adjusted for first on-site IPC visit timing. We also assessed within-facility correlation using facility-clustered standard errors, generalized estimating equations, and mixed-effects negative binomial models. All analyses were performed in R version 4.5.1 (R Foundation for Statistical Computing, Vienna, Austria). All tests were two-sided, and *P* < 0.05 was considered statistically significant.

## Results

### Descriptive characteristics

We analyzed 707 COVID-19 outbreaks that occurred in long-term care and other social welfare facilities in Okinawa Prefecture between April and September 2022. There were 338 (47.8%) outbreaks with a staff index case and 369 (52.2%) with a resident or non-staff index case. In the analytic data set overall, the median outbreak size was seven cases (IQR 2-18), and the mean was 14.4 cases. The analytic cohort included 128 (18.1%) outbreaks with a final outbreak size of one case, indicating that small outbreak episodes were also captured within the support system.

Screening was performed on the same day in 112 (15.8%) outbreaks, 1 day later in 204 (28.9%), 2 days later in 200 (28.3%), and ≥3 days later in 191 (27.0%) (Table 1). Among the 191 outbreaks in the ≥3-day category, the median delay was 4 days (IQR 3-5 days; range 3-17 days). Screening was performed on day 3 in 86 (45.0%) outbreaks and on day 4 in 54 (28.3%); the distribution is shown in Supplementary Table S8.

**Table 1:** Characteristics of COVID-19 outbreaks (N = 707).

(a) Delay from outbreak recognition to first unit-wide screening		
Delay to first unit-wide screening	n	% of outbreaks
Same-day	112	15.8
Next day	204	28.9
2 days later	200	28.3
≥3 days later	191	27.0
(b) Index case type		
Index case type	n	%
Staff index case	338	47.8
Resident or non-staff index case	369	52.2
(c) Facility type		
Facility type	n	%
Fee-based homes for older adults	315	44.6
Special nursing homes for the elderly	112	15.8
Long-term care health facilities	54	7.6
Service housing for older people	34	4.8
Other small elderly facilities^a^	94	13.3
Other or unknown facilities^b^	98	13.9
(d) Resident vaccination coverage		
Resident vaccination coverage	n	%
≥80%	240	33.9
50%-79%	34	4.8
<50%	12	1.7
Unknown/not ascertained	421	59.5
(e) Month of outbreak recognition		
Month of recognition	n	%
April 2022	89	12.6
May 2022	138	19.5
June 2022	100	14.1
July 2022	201	28.4
August 2022	150	21.2
September 2022	29	4.1
(f) On-site support visits		
Timing of first on-site support visit	n	%
No on-site visit	183	25.9
0-1 days after recognition	127	18.0
≥2 days after recognition	144	20.4
Unknown/not ascertained	253	35.8

Abbreviation: COVID-19, coronavirus disease 2019.

a Other small elderly facilities include group homes for older adults, small-scale multifunctional in-home care facilities, and care houses.

b Other or unknown facilities include disability facilities, daycare facilities, records coded as “other,” and outbreaks with missing facility type.

Outbreaks were most frequently recognized during July to August, corresponding to the large “seventh wave” of COVID-19 in Okinawa [6]. Fee-based homes for older adults and a heterogeneous group of other or unknown facilities (including disability welfare facilities) accounted for most outbreaks, whereas special nursing homes and long-term care health facilities contributed smaller proportions. Resident vaccination coverage was ≥80% in 240 (33.9%) outbreaks and was unknown or not ascertained in 421 (59.5%). Missing information was present in all facility categories, ranging from 50.0% in service housing for older people to 72.2% in long-term care health facilities, with modest evidence of variation by facility type (χ²[5] = 11.30, *P* = 0.046) (Supplementary Table S9). No on-site support visit was recorded for 183 outbreaks (25.9%), whereas the timing of the first on-site support visit was unknown or not ascertained for 253 (35.8%) outbreaks.

### Outbreak size by screening delay and index case type

Outbreak size tended to increase with longer delays to screening in both index case groups, and outbreaks with staff index cases were generally smaller than those with resident or non-staff index cases (Table 2, Figure 1). Among staff index outbreaks, the median outbreak size increased from two cases (IQR 1.0-12.0) with same-day screening to nine cases (IQR 3.0-32.5) when screening was delayed by ≥3 days. Among outbreaks with a resident or non-staff index case, the median outbreak size was eight cases (IQR 1.5-14.5) with same-day screening and nine to 10 cases with delays of ≥2 days. Differences between same-day and next-day screening were modest, whereas outbreak size tended to be larger, with delays of ≥2 days, particularly when the index case was a staff member.

**Table 2:** Outbreak size (total infected residents and staff per outbreak) by delay from outbreak recognition to unit-wide screening and index case type, Okinawa Prefecture, April to September 2022.

Delay from recognition to screening	Index case type	No. of outbreaks	Median outbreak size (IQR)	Mean outbreak size
Same day	Staff	69	2.0 (1.0-12.0)	10.1
	Resident or non-staff	43	8.0 (1.5-14.5)	11.6
Next day	Staff	95	4.0 (1.0-9.5)	8.7
	Resident or non-staff	109	7.0 (4.0-18.0)	12.7
2 days later	Staff	80	4.5 (1.0-19.2)	14.0
	Resident or non-staff	120	10.0 (4.0-24.2)	16.0
≥3 days later	Staff	94	9.0 (3.0-32.5)	20.7
	Resident or non-staff	97	9.0 (4.0-23.0)	18.2

Abbreviation: IQR, interquartile range.

**Figure 1:**
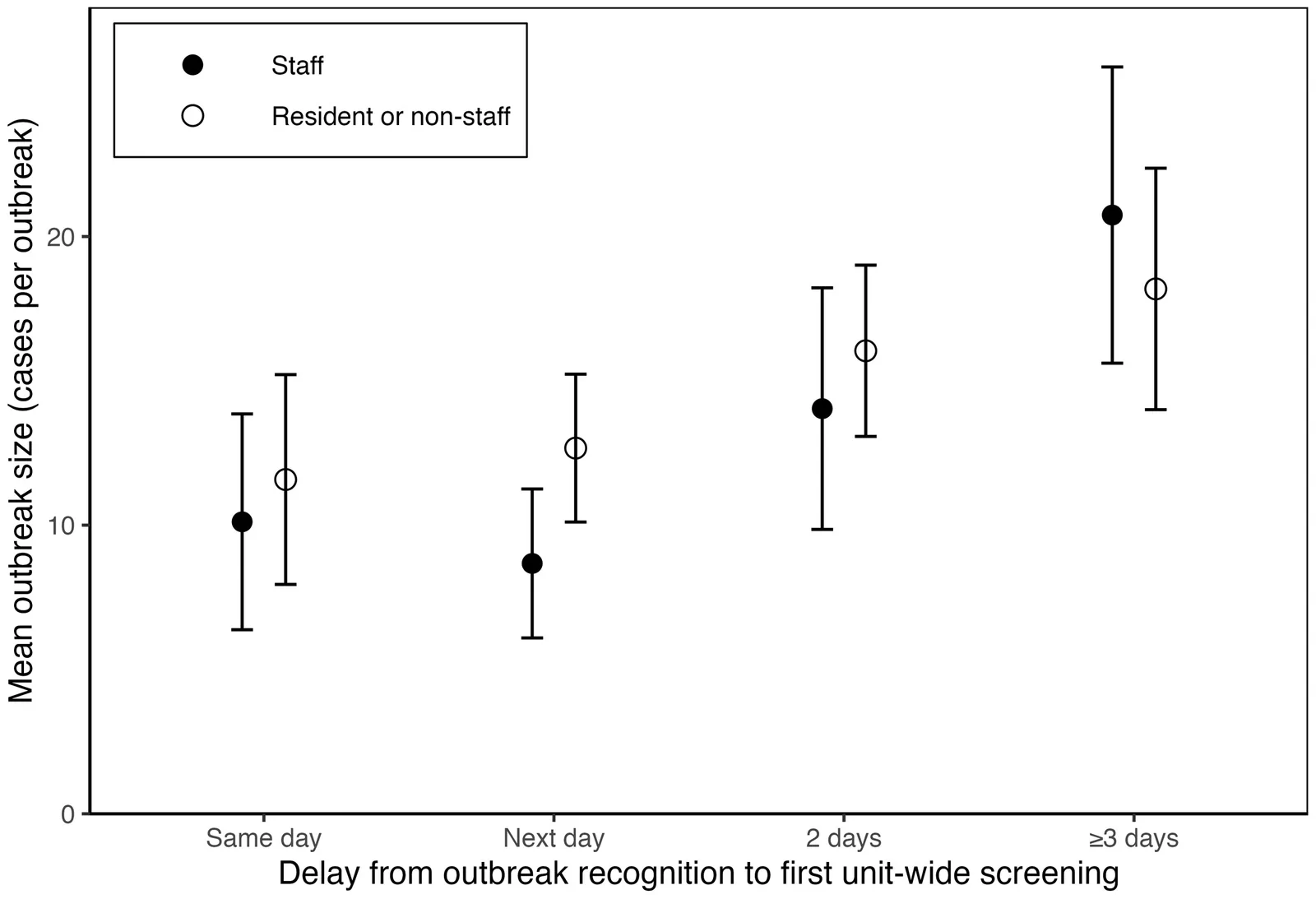
Mean outbreak size by delay to first unit-wide screening after outbreak recognition, stratified by index case type. Points indicate the mean final outbreak size, and error bars indicate 95% confidence intervals. Delay was categorized as the same day, the next day, 2 days later, or ≥3 days later. Final outbreak size was defined as the total number of laboratory-confirmed COVID-19 cases among residents and staff in each outbreak, including the index case. The resident or non-staff index case group included residents, day-service users, family members, and episodes with an unknown route of introduction. Abbreviation: COVID-19, coronavirus disease 2019.

### Multivariable association between screening delay and outbreak size

Univariate negative binomial regression results are presented in Supplementary Table S4. In the primary multivariable negative binomial regression model (Table 3), delay from outbreak recognition to the first unit-wide screening was associated with final outbreak size after adjustment for index case type, facility type, month of outbreak recognition, and resident vaccination coverage. Using same-day screening as the reference, adjusted incidence rate ratios (aIRRs) for total outbreak size were 0.95 (95% CI 0.72-1.26) for 1-day, 1.16 (95% CI 0.87-1.54) for 2-day, and 1.44 (95% CI 1.09-1.91) for ≥3-day delays. Thus, a delay of at least 3 days was associated with significantly larger outbreaks than same-day screening, whereas the CIs for the 1-day and 2-day categories included 1.

**Table 3:** Negative binomial regression of factors associated with outbreak size (total infected residents and staff per outbreak), Okinawa Prefecture, April to September 2022.

Exposure	Category (vs reference)	No. of outbreaks	aIRR (95% CI)	*P*-value
Delay to first unit-wide screening				
	Same day	112	1.00 (reference)	
	Next day	204	0.95 (0.72-1.26)	0.74
	2 days later	200	1.16 (0.87-1.54)	0.32
	≥3 days later	191	1.44 (1.09-1.91)	0.01*
Index case type				
	Staff	338	1.00 (reference)	
	Resident or non-staff	369	1.23 (1.03-1.48)	0.02*
Facility type				
	Fee-based homes for older adults	315	1.00 (reference)	
	Special nursing homes for the elderly	112	1.18 (0.91-1.53)	0.20
	Long-term care health facilities	54	1.88 (1.36-2.58)	<0.001***
	Service housing for older people	34	0.92 (0.61-1.39)	0.69
	Other small elderly facilities^a^	94	0.47 (0.36-0.61)	<0.001***
	Other or unknown facilities^b^	98	1.05 (0.78-1.41)	0.74
Month of recognition				
	April-May 2022	227	1.00 (reference)	
	June-July 2022	301	1.14 (0.89-1.45)	0.30
	August-September 2022	179	0.80 (0.61-1.06)	0.12
Resident vaccination coverage				
	≥80%	240	1.00 (reference)	
	50%-79%	34	1.30 (0.92-1.84)	0.14
	<50%	12	0.75 (0.48-1.17)	0.20
	Unknown	421	0.57 (0.46-0.70)	<0.001***

Abbreviations: aIRR, adjusted incidence rate ratio; CI, confidence interval.

Model adjusted simultaneously for all variables shown above.

Reference categories: same-day screening, staff index case, fee-based homes for older adults, April to May 2022, and resident vaccination coverage ≥80%.

All aIRRs are adjusted for the other variables in the table. Robust (HC1) standard errors are reported.

a Other small elderly facilities include group homes for older adults, small-scale multifunctional in-home care facilities, and care houses.

b Other or unknown facilities include disability facilities, daycare facilities, records coded as “other,” and outbreaks with missing facility type. *P*-value symbols: *** *P* < 0.001; * *P* < 0.05

Outbreaks with a resident or non-staff index case were larger than those with a staff index case (aIRR 1.23, 95% CI 1.03-1.48). Relative to fee-based homes for older adults, long-term care health facilities had substantially larger outbreaks (aIRR 1.88, 95% CI 1.36-2.58) and other small elderly facilities had smaller outbreaks (aIRR 0.47, 95% CI 0.36-0.61). There was no consistent gradient across categories of resident vaccination coverage; facilities with unknown coverage had smaller outbreaks on average (aIRR 0.57, 95% CI 0.46-0.70), although the interpretation of this variable was limited by substantial missingness in the operational records and the estimate for the unknown category may reflect differences in facility type, outbreak reporting, and data recording rather than a biologically meaningful protective association.

### Sensitivity analyses

Sensitivity analyses using alternative month and facility type categories yielded similar estimates for screening delay (Supplementary Tables S1 and S5). Estimates were also similar across approaches that accounted for within-facility correlation, including facility-clustered standard errors, generalized estimating equations, and mixed-effects negative binomial models (Supplementary Table S7). When additionally adjusting for the timing of the first on-site IPC visit, the association for screening delays of ≥3 days was attenuated and was no longer statistically significant. The aIRR for delays of ≥3 days decreased from 1.44 in the primary model to 1.18, with the CI including 1 (Supplementary Table S3). In the model that included an offset based on 1.5 × category-specific average licensed capacity, the screening-delay estimates were unchanged (Supplementary Table S2). A summary of the screening-delay estimates across the primary and sensitivity models is provided in Supplementary Table S6.

## Discussion

In 707 supported outbreaks, delays of ≥3 days from recognition to first unit-wide screening were associated with larger final outbreak size, whereas 1- to 2-day delays were not clearly associated after adjustment. These findings suggest that, within this supported outbreak-response system, a prolonged delay to outbreak-triggered screening may mark a higher-risk outbreak-response pattern in LTCFs and related social welfare facilities. However, because this was an observational study and the outcome was an absolute case count without facility-level denominators, the findings should be interpreted as associations rather than causal effects.

These findings are consistent with previous evidence that timely outbreak testing may support control in LTCFs [5,[7], [8], [9], [10], [11], [12], [13], [14], [15], [16]]. During Omicron, shorter incubation and serial intervals may have narrowed the practical window for screening after recognition [20,21]. However, the ≥3-day association should not be interpreted as a universal biological or operational threshold. Additional adjustment for on-site IPC visit timing attenuated the ≥3-day estimate from 1.44 to 1.18. Because visit timing may reflect outbreak complexity and response intensity, this attenuation further supports cautious interpretation of the primary association.

An interpretive limitation is that the exposure was defined by operational outbreak recognition rather than by the unobservable time of the first within-facility transmission. Although facilities were expected to notify the support team when within-facility spread was suspected, the analytic data set did not contain consistent measures of outbreak size or complexity at recognition. Outbreaks that were already larger or more complex at recognition may have required more time to organize unit-wide screening and may also have resulted in larger final outbreak sizes. We could not directly assess or adjust for this possibility. Thus, reverse causation or residual confounding by outbreak stage and complexity at recognition may have contributed to the observed association. Accordingly, the observed association should be interpreted as relating to timeliness after operational recognition rather than to time from true outbreak onset alone and should not be interpreted as establishing a causal effect of screening delay on outbreak size.

A second important limitation is the lack of facility-level denominators. Larger facilities may accumulate more cases and require more time to organize unit-wide screening, resulting in residual confounding. Adjustment for facility type does not fully capture facility or affected-unit size. Although the offset analysis using category-specific average licensed capacity produced similar screening-delay estimates (Supplementary Table S2), this proxy did not capture facility-specific populations at risk.

Generalizability is also limited by selection into the analytic sample and by the local outbreak-response context. The present analysis was limited to episodes captured by the prefectural support system with documented unit-wide screening. Although we could not formally quantify the coverage of all facility outbreaks in Okinawa, the analytic cohort included 128 (18.1%) outbreaks with a final size of one case, indicating that capture was not limited to larger outbreaks. The findings apply most directly to supported outbreaks with documented unit-wide screening and may not fully generalize to settings with substantially different testing access, staffing, or IPC infrastructure. In addition, because the exposure depended on the documented date of first unit-wide screening, any discrepancy between implementation and documentation could introduce information bias.

Interpretation of some covariates was also limited by data quality. In particular, resident vaccination coverage was unavailable for a substantial proportion of outbreaks, with the proportion of missing information varying across facility types. Missingness was nevertheless present in all facility categories rather than being confined to a single facility type. The coefficient for the “unknown” category may reflect differences in documentation practices or facility reporting processes and other unmeasured facility characteristics rather than a biologically meaningful association. The cross-tabulation describes the distribution of missingness but does not establish its mechanism or explain the lower adjusted estimate.

Similarly, we could not distinguish the operational causes of delayed screening, such as staffing shortages; limitations in specimen collection or transport capacity; and competing demands on facilities, public health centers, or external support teams. In addition, the timing and scope of outbreak-related testing varied according to the clinical and epidemiologic situation. Unit-wide screening could be undertaken from the outset when an outbreak was strongly suspected or was introduced later as further evidence supported broader testing. A longer interval may, therefore, have reflected decisions about the scope of testing and practical constraints on implementation rather than simply a delay in implementing a predetermined unit-wide screening plan. More complex outbreaks may also have required greater logistical coordination for broader testing. These factors were not consistently recorded and may have been associated with screening delay and final outbreak size, resulting in unmeasured confounding.

Routine staff RT-PCR screening may also have influenced outbreak ascertainment. A positive routine screening result could have prompted recognition at an earlier stage, whereas recent testing of some staff could have influenced the timing or scope of subsequent outbreak-response testing. Information on index case detection pathways and recent routine screening dates was not consistently available at the episode level in the analytic data set. We, therefore, could not quantify this overlap or account for its potential influence on outbreak stage at recognition and the observed association between screening delay and outbreak size.

These findings complement our earlier questionnaire study, which examined detection and initial response practices rather than screening timing [6]. Together, the studies suggest that early detection and timely post-recognition screening may be relevant during high community transmission, although both are observational.

## Conclusion

Among COVID-19 outbreaks in long-term care and related social welfare facilities in Okinawa Prefecture that were recorded in the prefectural outbreak-response records, delays of ≥3 days from outbreak recognition to the first unit-wide screening were associated with larger final outbreak size. These findings suggest that screening timeliness may be relevant to outbreak control in similar LTCFs and related social welfare facilities while recognizing that causal inference is limited by the observational design, unavailable facility-level denominators, residual confounding, and possible differences in outbreak stage at recognition.

## Funding

This work was supported by JSPS KAKENHI (20H03940, 20KK0367, 26K02652 and 21K10416) and JST SICORP (JPMJSC21U4). The funders had no role in study design, data collection, analysis, interpretation, manuscript preparation, or the decision to submit the article.

## Ethical approval

This study was a secondary analysis of anonymized outbreak-support data collected by Okinawa Prefecture. The data set contained no information that could directly or indirectly identify individuals. Analyses were conducted in accordance with applicable Japanese law and Okinawa Prefectural Government policy and were exempt from institutional review board review.

## Data availability

The data that support the findings of this study are not publicly available because they were used under license and contain information subject to restrictions by the Okinawa Prefectural Government. Data may be available from the authors upon reasonable request and with permission from the Okinawa Prefectural Government.

## Consent for publication

Not applicable.

## Declaration of generative AI and AI-assisted technologies in the writing process

During the preparation of this manuscript, the authors used ChatGPT (OpenAI) to assist in improving language and readability. After using this tool, the authors reviewed and edited the content as needed and take full responsibility for the content of the publication.

## CRediT authorship contribution statement

**Yining S. Xu:** Writing – original draft, Visualization, Validation, Software, Formal analysis, Data curation. **Kuniko Takahashi:** Writing – review & editing. **Yusuke Shimakawa:** Writing – review & editing. **Takashi Nakamura:** Writing – review & editing, Resources, Investigation. **Toru Itokazu:** Writing – review & editing, Resources. **Yoshihiro Takayama:** Writing – review & editing, Project administration, Formal analysis, Conceptualization. **Kenji Mizumoto:** Writing – original draft, Supervision, Software, Project administration, Methodology, Funding acquisition, Formal analysis, Conceptualization.

## Declaration of competing interest

The authors have no competing interests to declare.

## Appendix Supplementary materials

Supplementary Tables S1-S9 accompany this article and provide additional details. Image, application 1

## References

[1] Henriques H.R., Sousa D., Faria J., Pinto J., Costa A., Henriques M.A. (2023). Learning from the COVID-19 outbreaks in long-term care facilities: a systematic review. BMC Geriatr.

[2] Dykgraaf S.H., Matenge S., Desborough J., Sturgiss E., Dut G., Roberts L. (2021). Protecting nursing homes and long-term care facilities from COVID-19: a rapid review of international evidence. J Am Med Dir Assoc.

[3] Iritani O., Okuno T., Hama D., Kane A., Kodera K., Morigaki K. (2020). Clusters of COVID-19 in long-term care hospitals and facilities in Japan from 16 January to 9 May 2020. Geriatr Gerontol Int.

[4] Yang H., Rigsby M., Zhu X., Lee C., Ory M. (2022). COVID-19 in long-term care facilities: a rapid review of infection correlates and impacts on mental health and behaviors. HERD.

[5] Gómez Vázquez J.P., García Y.E., Schmidt A.J., Martínez-López B., Nuño M. (2022). Testing and vaccination to reduce the impact of COVID-19 in nursing homes: an agent-based approach. BMC Infect Dis.

[6] Xu Y.S., Shimakawa Y., Chowell G., Matsuyama R., Nagamoto T., Omori R. (2026). Determinants of COVID-19 outbreak size in elderly residential facilities in Okinawa Prefecture, Japan, April to June 2022. IJID Reg.

[7] Arons M.M., Hatfield K.M., Reddy S.C., Kimball A., James A., Jacobs J.R. (2020). Presymptomatic SARS-CoV-2 infections and transmission in a skilled nursing facility. N Engl J Med.

[8] Kimball A., Hatfield K.M., Arons M., James A., Taylor J., Spicer K. (2020). Asymptomatic and presymptomatic SARS-CoV-2 infections in residents of a long-term care skilled nursing facility—King County, Washington, March 2020. MMWR Morb Mortal Wkly Rep.

[9] McMichael T.M., Currie D.W., Clark S., Pogosjans S., Kay M., Schwartz N.G. (2020). Epidemiology of COVID-19 in a long-term care facility in King County, Washington. N Engl J Med.

[10] Mizumoto K., Kagaya K., Zarebski A., Chowell G. (2020). Estimating the asymptomatic proportion of coronavirus disease 2019 (COVID-19) cases on board the Diamond Princess cruise ship, Yokohama, Japan, 2020. Euro Surveill.

[11] Dora A.V., Winnett A., Jatt L.P., Davar K., Watanabe M., Sohn L. (2020). Universal and serial laboratory testing for SARS-CoV-2 at a long-term care skilled nursing facility for veterans—Los Angeles, California, 2020. MMWR Morb Mortal Wkly Rep.

[12] Hatfield K.M., Reddy S.C., Forsberg K., Korhonen L., Garner K., Gulley T. (2020). Facility-wide testing for SARS-CoV-2 in nursing homes—seven U.S. jurisdictions, March–June 2020. MMWR Morb Mortal Wkly Rep.

[13] Telford C.T., Onwubiko U., Holland D.P., Turner K., Prieto J., Smith S. (2020). Preventing COVID-19 outbreaks in long-term care facilities through preemptive testing of residents and staff members—Fulton County, Georgia, March–May 2020. MMWR Morb Mortal Wkly Rep.

[14] Bigelow B.F., Tang O., Barshick B., Peters M., Sisson S.D., Peairs K.S. (2021). Outcomes of universal COVID-19 testing following detection of incident cases in 11 long-term care facilities. JAMA Intern Med.

[15] Smith D.R.M., Duval A., Pouwels K.B., Guillemot D., Fernandes J., Huynh B.T. (2020). Optimizing COVID-19 surveillance in long-term care facilities: a modelling study. BMC Med.

[16] See I., Paul P., Slayton R.B., Steele M.K., Stuckey M.J., Duca L. (2021). Modeling effectiveness of testing strategies to prevent coronavirus disease 2019 (COVID-19) in nursing homes-United States, 2020. Clin Infect Dis.

[17] National Center for Immunization and Respiratory Diseases (NCIRD), Division of Viral Diseases, Centers for Disease Control and Prevention (2022). Interim infection prevention and control recommendations to prevent SARS-CoV-2 spread in nursing homes: nursing homes & long-term care facilities. https://stacks.cdc.gov/view/cdc/114001.

[18] Brown K.A., Buchan S.A., Chan A.K., Costa A., Daneman N., Garber G. (2024). Association between delayed outbreak identification and SARS-CoV-2 infection and mortality among long-term care home residents, Ontario, Canada, March to November 2020: a cohort study. Euro Surveill.

[19] Department of Infectious Diseases, Okinawa Prefectural Chubu Hospital (2021).

[20] Wu Y., Kang L., Guo Z., Liu J., Liu M., Liang W. (2022). Incubation period of COVID-19 caused by unique SARS-CoV-2 strains: a systematic review and meta-analysis. JAMA Netw Open.

[21] Zeng K., Santhya S., Soong A., Malhotra N., Pushparajah D., Thoon K.C. (2023). Serial intervals and incubation periods of SARS-CoV-2 omicron and delta variants, Singapore. Emerg Infect Dis.

