# Supplementary material for: Timing of outbreak-triggered unit-wide screening and COVID-19 outbreak size in long-term care and related social welfare facilities in Okinawa, Japan
Source: IJID Reg. 2026 Oct 13;21:101042. doi: 10.1016/j.ijregi.2026.101042 (PMC13744804; doi:10.1016/j.ijregi.2026.101042)
Supplement: Supplementary file 1 [file mmc1.docx]

**Supplementary information**

Timing of outbreak-triggered unit-wide screening and COVID-19 outbreak size in long-term care and related social welfare facilities in Okinawa, Japan

**Supplementary information:**

The electronic supplementary material is below the table titles.

Supplementary Tables S1–S9 provide supporting data and model results referenced in the main text.

**Table S1. Negative binomial regression of factors associated with outbreak size (total infected residents and staff per outbreak), using a six-category month variable and six-category facility type, Okinawa Prefecture, April–September 2022.**

| Exposure | Category (vs reference) | No. of outbreaks | aIRR (95% CI) | p-value |
| --- | --- | --- | --- | --- |
| Delay to first unit-wide screening | |  |  |  |
|  | Same-day | 112 | 1.00 (reference) |  |
|  | Next day | 204 | 0.96 (0.72–1.27) | 0.76 |
|  | 2 days later | 200 | 1.13 (0.85–1.51) | 0.41 |
|  | ≥3 days later | 191 | 1.37 (1.04–1.82) | 0.03* |
| Index case type | |  |  |  |
|  | Staff | 338 | 1.00 (reference) |  |
|  | Resident or non-staff | 369 | 1.24 (1.03–1.49) | 0.02* |
| Facility type | |  |  |  |
|  | Fee-based homes for older adults | 315 | 1.00 (reference) |  |
|  | Special nursing homes for the elderly | 112 | 1.17 (0.90–1.52) | 0.23 |
|  | Long-term care health facilities | 54 | 1.78 (1.29–2.45) | <0.001*** |
|  | Service housing for older people | 34 | 0.91 (0.60–1.39) | 0.66 |
|  | Other small elderly facilities† | 94 | 0.47 (0.36–0.61) | <0.001*** |
|  | Other or unknown facilities^§^ | 98 | 1.01 (0.76–1.36) | 0.92 |
| Month of recognition | |  |  |  |
|  | Apr 2022 | 89 | 1.00 (reference) |  |
|  | May 2022 | 138 | 1.09 (0.76–1.58) | 0.63 |
|  | Jun 2022 | 100 | 1.05 (0.71–1.56) | 0.81 |
|  | Jul 2022 | 201 | 1.40 (0.97–2.03) | 0.07 . |
|  | Aug 2022 | 150 | 1.04 (0.71–1.51) | 0.85 |
|  | Sep 2022 | 29 | 0.52 (0.31–0.87) | 0.01 * |
| Resident vaccination coverage | |  |  |  |
|  | ≥80% | 240 | 1.00 (reference) |  |
|  | 50–79% | 34 | 1.29 (0.91–1.81) | 0.15 |
|  | <50% | 12 | 0.82 (0.52–1.27) | 0.37 |
|  | Unknown | 421 | 0.64 (0.51–0.81) | <0.001*** |
| aIRR: Adjusted incidence rate ratio; CI: confidence interval. Model adjusted simultaneously for all variables shown above. | | | | |
| Reference categories: same-day screening, staff index case, fee-based homes for older adults, April 2022, and resident vaccination coverage ≥80%. | | | | |
| ^§^ Other or unknown facilities include disability facilities, daycare facilities, records coded as “other”, and outbreaks with missing facility type. | | | | |
| † Other small elderly facilities include group homes for older adults, small-scale multifunctional in-home care facilities, and care houses. | | | | |
| All aIRRs are adjusted for the other variables in the table. Robust (HC1) standard errors are reported. | | | | |
| p-value symbols: *** p<0.001; ** p<0.01; * p<0.05; . p<0.1 | | | | |

**Table S2.** **Negative binomial regression of outbreak size including a log-offset for 1.5 × category-specific average licensed capacity, Okinawa Prefecture, April–September 2022.**

| Exposure | Category (vs reference) | No. of outbreaks | aIRR (95% CI) | p-value |
| --- | --- | --- | --- | --- |
| Delay to first unit-wide screening | | |  |  |
|  | Same-day | 112 | 1.00 (reference) |  |
|  | Next day | 204 | 0.95 (0.72–1.26) | 0.74 |
|  | 2 days later | 200 | 1.16 (0.87–1.54) | 0.32 |
|  | ≥3 days later | 191 | 1.44 (1.09–1.91) | 0.01* |
| Index case type | |  |  |  |
|  | Staff | 338 | 1.00 (reference) |  |
|  | Resident or non-staff | 369 | 1.23 (1.03–1.48) | 0.02* |
| Facility type | |  |  |  |
|  | Fee-based homes for older adults | 315 | 1.00 (reference) |  |
|  | Special nursing homes for the elderly | 112 | 0.44 (0.34–0.56) | <0.001*** |
|  | Long-term care health facilities | 54 | 0.49 (0.36–0.68) | <0.001*** |
|  | Service housing for older people | 34 | 0.65 (0.43–0.98) | 0.04* |
|  | Other small elderly facilities† | 94 | 0.39 (0.30–0.50) | <0.001*** |
|  | Other or unknown facilities^§^ | 98 | 0.60 (0.45–0.80) | <0.001*** |
| Month of recognition | |  |  |  |
|  | April–May 2022 | 227 | 1.00 (reference) |  |
|  | June–July 2022 | 301 | 1.14 (0.89–1.45) | 0.30 |
|  | August–September 2022 | 179 | 0.80 (0.61–1.06) | 0.12 |
| Resident vaccination coverage | | |  |  |
|  | ≥80% | 240 | 1.00 (reference) |  |
|  | 50–79% | 34 | 1.30 (0.92–1.84) | 0.14 |
|  | <50% | 12 | 0.75 (0.48–1.17) | 0.20 |
|  | Unknown | 421 | 0.57 (0.46–0.70) | <0.001*** |
| aIRR: Adjusted incidence rate ratio; CI: confidence interval. Model adjusted simultaneously for all variables shown above. | | | | |
| Reference categories: same-day screening, staff index case, fee-based homes for older adults, April–May 2022, and resident vaccination coverage ≥80%. | | | | |
| ^§^ Other or unknown facilities include disability facilities, daycare facilities, records coded as “other”, and outbreaks with missing facility type. | | | | |
| † Other small elderly facilities include group homes for older adults, small-scale multifunctional in-home care facilities, and care houses. | | | | |
| The log-offset was specified as log(1.5 × category-specific average licensed capacity), with the factor 1.5 used to approximate the total population at risk. Category-specific average licensed capacities for each facility-type group were derived from the 2024 Okinawa Prefecture social welfare facility directory (R6 social welfare facility directory, Okinawa) [1]. These values do not capture facility-specific variation in the number of residents and staff at risk. Because facility type was also included as a covariate, this analysis should not be interpreted as an independent adjustment for actual facility size. | | | | |
| p-value symbols: *** p<0.001; ** p<0.01; * p<0.05; . p<0.1 | | | | |

**Table S3.** **Negative binomial regression of outbreak size additionally adjusting for the timing of the first on-site IPC visit, Okinawa Prefecture, April–September 2022.**

| Exposure | Category (vs reference) | No. of outbreaks | aIRR (95% CI) | p-value |
| --- | --- | --- | --- | --- |
| Delay to first unit-wide screening | |  |  |  |
|  | Same-day | 112 | 1.00 (reference) |  |
|  | Next day | 204 | 0.89 (0.68–1.17) | 0.41 |
|  | 2 days later | 200 | 1.02 (0.77–1.36) | 0.88 |
|  | ≥3 days later | 191 | 1.18 (0.91–1.54) | 0.22 |
| Index case type | |  |  |  |
|  | Staff | 338 | 1.00 (reference) |  |
|  | Resident or non-staff | 369 | 1.12 (0.93–1.35) | 0.24 |
| Facility type | |  |  |  |
|  | Fee-based homes for older adults | 315 | 1.00 (reference) |  |
|  | Special nursing homes for the elderly | 112 | 1.39 (1.07–1.80) | 0.01* |
|  | Long-term care health facilities | 54 | 2.15 (1.58–2.92) | <0.001*** |
|  | Service housing for older people | 34 | 0.81 (0.61–1.09) | 0.16 |
|  | Other small elderly facilities† | 94 | 0.61 (0.47–0.80) | <0.001*** |
|  | Other or unknown facilities^§^ | 98 | 1.02 (0.79–1.32) | 0.89 |
| Month of recognition | |  |  |  |
|  | April–May 2022 | 227 | 1.00 (reference) |  |
|  | June–July 2022 | 301 | 1.29 (1.00–1.67) | 0.05 . |
|  | August–September 2022 | 179 | 0.95 (0.70–1.29) | 0.75 |
| Resident vaccination coverage | |  |  |  |
|  | ≥80% | 240 | 1.00 (reference) |  |
|  | 50–79% | 34 | 1.31 (0.85–2.02) | 0.22 |
|  | <50% | 12 | 0.77 (0.53–1.13) | 0.19 |
|  | Unknown | 421 | 0.69 (0.56–0.85) | <0.001*** |
| On-site support visit | |  |  |  |
|  | No on-site visit | 183 | 1.00 (reference) |  |
|  | 0–1 days after recognition | 127 | 4.37 (3.20–5.95) | <0.001*** |
|  | ≥2 days after recognition | 144 | 4.10 (3.06–5.50) | <0.001*** |
|  | Unknown / not ascertained | 253 | 2.13 (1.55–2.93) | <0.001*** |
| aIRR: Adjusted incidence rate ratio; CI: confidence interval. Model adjusted simultaneously for all variables shown above. | | | | |
| Reference categories: same-day screening, no on-site support visit, staff index case, fee-based homes for older adults, April–May 2022, and resident vaccination coverage ≥80%. | | | | |
| ^§^ Other or unknown facilities include disability facilities, daycare facilities, records coded as “other”, and outbreaks with missing facility type. | | | | |
| † Other small elderly facilities include group homes for older adults, small-scale multifunctional in-home care facilities, and care houses. | | | | |
| All aIRRs are adjusted for the other variables in the table. Robust (HC1) standard errors are reported. | | | | |
| Significance symbols are based on unrounded p-values. | | | | |
| p-value symbols: *** p<0.001; ** p<0.01; * p<0.05; . p<0.1 | | | | |

**Table S4.** **Univariate negative binomial regression of factors associated with COVID-19 outbreak size in long-term care and social welfare facilities, Okinawa Prefecture, April–September 2022.**

| Exposure | Category (vs reference) | No. of outbreaks | Unadjusted IRR (95% CI) | p-value |
| --- | --- | --- | --- | --- |
| Delay to first unit-wide screening | | |  |  |
|  | Same-day | 112 | 1.00 (reference) |  |
|  | Next day | 204 | 1.01 (0.75–1.37) | 0.94 |
|  | 2 days later | 200 | 1.43 (1.06–1.92) | 0.02* |
|  | ≥3 days later | 191 | 1.82 (1.34–2.47) | <0.001*** |
| Index case type | |  |  |  |
|  | Staff | 338 | 1.00 (reference) |  |
|  | Resident or non-staff | 369 | 1.11 (0.92–1.34) | 0.28 |
| Facility type | |  |  |  |
|  | Fee-based homes for older adults | 315 | 1.00 (reference) |  |
|  | Special nursing homes for the elderly | 112 | 1.25 (0.96–1.63) | 0.10 |
|  | Long-term care health facilities | 54 | 1.91 (1.40–2.62) | <0.001*** |
|  | Service housing for older people | 34 | 0.95 (0.66–1.37) | 0.78 |
|  | Other small elderly facilities† | 94 | 0.44 (0.34–0.57) | <0.001*** |
|  | Other or unknown facilities^§^ | 98 | 1.09 (0.80–1.49) | 0.57 |
| Month of recognition | |  |  |  |
|  | April–May 2022 | 227 | 1.00 (reference) |  |
|  | June–July 2022 | 301 | 1.95 (1.57–2.43) | <0.001*** |
|  | August–September 2022 | 179 | 1.70 (1.34–2.17) | <0.001*** |
| Resident vaccination coverage | | |  |  |
|  | ≥80% | 240 | 1.00 (reference) |  |
|  | 50–79% | 34 | 1.31 (0.93–1.85) | 0.12 |
|  | <50% | 12 | 0.68 (0.43–1.07) | 0.10 |
|  | Unknown | 421 | 0.56 (0.46–0.68) | <0.001*** |
| On-site support visit | |  |  |  |
|  | No on-site visit | 183 | 1.00 (reference) |  |
|  | 0–1 days after recognition | 127 | 4.79 (3.63–6.34) | <0.001*** |
|  | ≥2 days after recognition | 144 | 5.70 (4.34–7.48) | <0.001*** |
|  | Unknown / not ascertained | 253 | 3.26 (2.45–4.33) | <0.001*** |
| IRR: Incidence rate ratio; CI: confidence interval. Each IRR is from a separate univariate negative binomial regression model including only the exposure shown. | | | | |
| Reference categories: same-day screening, no on-site support visit, staff index case, fee-based homes for older adults, April–May 2022, and resident vaccination coverage ≥80%. | | | | |
| ^§^ Other or unknown facilities include disability facilities, daycare facilities, records coded as “other”, and outbreaks with missing facility type. | | | | |
| † Other small elderly facilities include group homes for older adults, small-scale multifunctional in-home care facilities, and care houses. | | | | |
| p-value symbols: *** p<0.001; ** p<0.01; * p<0.05; . p<0.1 | | | | |

**Table S5. Negative binomial regression of outbreak size in a model treating disability welfare facilities as an independent facility type category, Okinawa Prefecture, April–September 2022.**

| Exposure | Category (vs reference) | No. of outbreaks | aIRR (95% CI) | p-value |
| --- | --- | --- | --- | --- |
| Delay to first unit-wide screening | |  |  |  |
|  | Same-day | 112 | 1.00 (reference) |  |
|  | Next day | 204 | 0.95 (0.72–1.26) | 0.73 |
|  | 2 days later | 200 | 1.16 (0.87–1.54) | 0.33 |
|  | ≥3 days later | 191 | 1.44 (1.08–1.92) | 0.01* |
| Index case type | |  |  |  |
|  | Staff | 338 | 1.00 (reference) |  |
|  | Resident or non-staff | 369 | 1.24 (1.03–1.48) | 0.02* |
| Facility type | |  |  |  |
|  | Fee-based homes for older adults | 315 | 1.00 (reference) |  |
|  | Special nursing homes for the elderly | 112 | 1.18 (0.91–1.53) | 0.20 |
|  | Long-term care health facilities | 54 | 1.88 (1.36–2.58) | <0.001*** |
|  | Service housing for older people | 34 | 0.92 (0.61–1.39) | 0.69 |
|  | Other small elderly facilities† | 94 | 0.47 (0.36–0.61) | <0.001*** |
|  | Disability welfare facilities | 74 | 1.07 (0.76–1.49) | 0.71 |
|  | Other or unknown facilities^§^ | 24 | 1.00 (0.57–1.78) | 0.99 |
| Month of recognition | |  |  |  |
|  | April–May 2022 | 227 | 1.00 (reference) |  |
|  | June–July 2022 | 301 | 1.14 (0.89–1.45) | 0.30 |
|  | August–September 2022 | 179 | 0.81 (0.61–1.06) | 0.12 |
| Resident vaccination coverage | |  |  |  |
|  | ≥80% | 240 | 1.00 (reference) |  |
|  | 50–79% | 34 | 1.30 (0.92–1.84) | 0.14 |
|  | <50% | 12 | 0.75 (0.48–1.17) | 0.20 |
|  | Unknown | 421 | 0.57 (0.46–0.70) | <0.001*** |
| aIRR: Adjusted incidence rate ratio; CI: confidence interval. Model adjusted simultaneously for all variables shown above. | | | | |
| Reference categories: same-day screening, staff index case, fee-based homes for older adults, April–May 2022, and resident vaccination coverage ≥80%. | | | | |
| ^§^ Other or unknown facilities include daycare facilities, records coded as “other”, and outbreaks with missing facility type. | | | | |
| † Other small elderly facilities include group homes for older adults, small-scale multifunctional in-home care facilities, and care houses. | | | | |
| All aIRRs are adjusted for the other variables in the table. Robust (HC1) standard errors are reported. | | | | |
| p-value symbols: *** p<0.001; ** p<0.01; * p<0.05; . p<0.1 | | | | |

**Table S6. Sensitivity analysis of the association between delay in unit-wide screening and outbreak size across multiple model specifications, Okinawa Prefecture, April–September 2022.**

| Sensitivity analysis | Model description | Adjusted IRR (95% CI) for outbreak size | | | | | |
| --- | --- | --- | --- | --- | --- | --- | --- |
|  |  | 1 day vs Same-day | p-value | 2 days vs Same-day | p-value | ≥3 days vs Same-day | p-value |
| Primary model | Primary model | 0.95 (0.72–1.26) | 0.74 | 1.16 (0.87–1.54) | 0.32 | 1.44 (1.09–1.91) | 0.01* |
| Model 1 | Six-category month model | 0.96 (0.72–1.27) | 0.76 | 1.13 (0.85–1.51) | 0.41 | 1.37 (1.04–1.82) | 0.03* |
| Model 2 | Offset model | 0.95 (0.72–1.26) | 0.74 | 1.16 (0.87–1.54) | 0.32 | 1.44 (1.09–1.91) | 0.01* |
| Model 3 | With IPC visit | 0.89 (0.68–1.17) | 0.41 | 1.02 (0.77–1.36) | 0.88 | 1.18 (0.91–1.54) | 0.22 |
| Model 4 | With disability facilities | 0.95 (0.72–1.26) | 0.73 | 1.16 (0.87–1.54) | 0.33 | 1.44 (1.08–1.92) | 0.01* |
| IRR: incidence rate ratio; CI: confidence interval; IPC: infection prevention and control. | | | | |  |  |  |
| Estimates are from negative binomial regression models with total outbreak size (infected residents and staff per outbreak) as the dependent variable. Each row corresponds to a separate multivariable model. | | | | | | | |
| Unless otherwise specified, all models were adjusted for index case type, facility type, month of outbreak recognition, and resident vaccination coverage, using the same reference categories as in the primary model (Table 3). The reference category for screening delay was same‑day screening. | | | | | | | |
| p-value symbols: *** p<0.001; ** p<0.01; * p<0.05; . p<0.1 | | | | | | | |

**Table S7. Robustness of the association between screening delay and outbreak size**

| Model | SE/Correlation | Adjusted IRR (95% CI) | | | | | |
| --- | --- | --- | --- | --- | --- | --- | --- |
|  |  | Next day | p-value | 2 days later | p-value | ≥3 days later | p-value |
| Primary NB | HC1 robust | 0.95 (0.72–1.26) | 0.74 | 1.16 (0.87–1.54) | 0.32 | 1.44 (1.09–1.91) | 0.01* |
| Sensitivity A | Cluster-robust (facility) | 0.95 (0.73–1.24) | 0.72 | 1.16 (0.88–1.52) | 0.30 | 1.44 (1.10–1.89) | <0.01** |
| Sensitivity B | GEE NB (exchangeable) | 0.97 (0.75–1.26) | 0.83 | 1.17 (0.89–1.54) | 0.27 | 1.43 (1.09–1.88) | <0.01** |
| Sensitivity C | Mixed-effects NB | 0.93 (0.71–1.22) | 0.62 | 1.13 (0.86–1.49) | 0.37 | 1.45 (1.10–1.90) | <0.01** |
| Estimates are adjusted incidence rate ratios (aIRRs) with 95% confidence intervals. The primary negative binomial model used heteroskedasticity-robust HC1 standard errors. Sensitivity analyses accounted for within-facility correlation using (i) facility-clustered standard errors, (ii) negative binomial generalized estimating equations (GEE) with an exchangeable working correlation structure, and (iii) a mixed-effects negative binomial model with a facility-specific random intercept. All models included index case type, facility type, month of outbreak recognition, and resident vaccination coverage. Additional adjustment for the timing of the first on-site IPC visit is presented separately in Table S3. | | | | | | | |
| p-value symbols: *** p<0.001; ** p<0.01; * p<0.05; . p<0.1 | | | | | | | |

Table S8. Distribution of screening delays in calendar days among outbreaks in the ≥3-day category, Okinawa Prefecture, April–September 2022.

| Delay from outbreak recognition to first unit-wide screening | n | % |
| --- | --- | --- |
| 3 days | 86 | 45.0 |
| 4 days | 54 | 28.3 |
| 5 days | 12 | 6.3 |
| 6 days | 9 | 4.7 |
| ≥7 days | 30 | 15.7 |
| Total | 191 | 100.0 |
| This table includes the 191 outbreaks in the ≥3-day category of the 707-outbreak analytic cohort. Delays were calculated in calendar days from the recorded dates of outbreak recognition and first unit-wide screening. Percentages use 191 as the denominator. The median delay was 4 days (IQR, 3–5 days; range, 3–17 days), and 140 (73.3%) were screened on days 3–4. IQR, interquartile range. | | |

Table S9. Resident vaccination coverage by facility type, Okinawa Prefecture, April–September 2022

| Facility type | Total outbreaks | ≥80%, n (%) | 50–79%, n (%) | <50%, n (%) | Unknown / not ascertained, n (%) |
| --- | --- | --- | --- | --- | --- |
| Fee-based homes for older adults | 315 | 116 (36.8) | 17 (5.4) | 9 (2.9) | 173 (54.9) |
| Special nursing homes for the elderly | 112 | 35 (31.2) | 6 (5.4) | 0 (0.0) | 71 (63.4) |
| Long-term care health facilities | 54 | 11 (20.4) | 4 (7.4) | 0 (0.0) | 39 (72.2) |
| Service housing for older people | 34 | 16 (47.1) | 1 (2.9) | 0 (0.0) | 17 (50.0) |
| Other small elderly facilities | 94 | 26 (27.7) | 4 (4.3) | 0 (0.0) | 64 (68.1) |
| Other or unknown facilities | 98 | 36 (36.7) | 2 (2.0) | 3 (3.1) | 57 (58.2) |
| All facility types | 707 | 240 (33.9) | 34 (4.8) | 12 (1.7) | 421 (59.5) |
| Values are numbers of outbreaks with row percentages in parentheses. Percentages may not sum to 100% because of rounding. Other small elderly facilities include group homes for older adults, small-scale multifunctional in-home care facilities, and care houses. Other or unknown facilities include disability welfare facilities, daycare facilities, records coded as “other”, and outbreaks with missing facility type. For assessment of missingness, resident vaccination coverage was dichotomized as known versus unknown/not ascertained. There was modest evidence of variation in missingness by facility type (Pearson’s χ²(5)=11.30, p=0.046). | | | | | |

# References

1. Okinawa Prefecture Council of Social Welfare. Okinawa social welfare facility directories [Internet]. Available from: https://www.okishakyo.or.jp/%E7%9C%8C%E5%86%85%E7%A6%8F%E7%A5%89%E6%96%BD%E8%A8%AD%E5%90%8D%E7%B0%BF/ [accessed 13 March 2026].
